# Cervix human papilloma virus positivity: Does it cause sexual dysfunction?

**DOI:** 10.4274/tjod.galenos.2019.18853

**Published:** 2020-02-28

**Authors:** Önder Sakin, Sakine Betül Uzun, Kazibe Koyuncu, Burak Giray, Emine Eda Akalın, Ali Doğukan Anğın

**Affiliations:** 1 University of Health Sciences, İstanbul Kartal Dr. Lütfi Kırdar Training and Research Hospital, Clinic of Obstetrics and Gynecology, İstanbul, Turkey; 2 University of Health Sciences, İstanbul Kartal Dr. Lütfi Kırdar Training and Research Hospital, Clinic of Family Medicine, İstanbul, Turkey

**Keywords:** Sexual dysfunction, physiological, human papilloma virus, cervix

## Abstract

**Objective:**

To investigate whether testing positive for human papilloma virus (HPV) in cervical screening has an impact on female sexual functioning.

**Materials and Methods:**

This study was designed as a single-center, prospective, descriptive-cross-sectional study and 300 women who received HPV testing in our hospital [HPV-positive (n=187) or HPV-negative (n=113)]. The Arizona Sexual Experiences (ASEX) scale and Female Sexual Functioning index (FSFI) were administered to study participants during face-to-face interviews.

**Results:**

No significant differences were found between women who were HPV-positive and HPV-negative in sexual functions as assessed using the ASEX and FSFI scales (p=0.343 and p=0.604, respectively). In addition, the analyses addressing whether sexual functioning was affected by a positive test result, at diagnosis or during the follow-up (before 2 weeks, 2 weeks-1 month, 1-3 months, 3-6 months, 6 months-1 year and over 1 year) revealed no significant differences between HPV-positive and HPV-negative women in sexual functioning (p>0.05). Sexual dysfunction was less common in married women than in the ASEX scale (p=0.03), and this difference was not detected when the FSFI scale was applied. The incidence of dysfunction was more frequent in working women than in retirees (p=0.006, p=0.01).

**Conclusion:**

Educational attainment, socioeconomic status, age, employment status, and marital status were found to have statistically significant effects on sexual functioning. Sexual functioning was affected by neither HPV test results (positive/negative) nor time from diagnosis.

**PRECIS:** Human papilloma virus results of the patients does not related to sexual dysfunction.

## Introduction

Female sexual function is defined as “the harmony in mind, senses and the individual’s body, which leads to the achievement of the personality, communication and love” (World Health Organization 2006)^(1)^. Female sexual dysfunction is described as libido abnormalities, stimulation and orgasm problems, along with sexual pain^(2)^.

In Turkey, the prevalence of sexual dysfunction in women has been reported to range between 46.9% and 48.3%^(3,4)^. Sexual function is affected by many factors such as menstruation, pregnancy, lactation, anogenital lesions, cancer, chronic systemic diseases, infertility, any conditions that might lead to sexual dysfunction including vaginismus, vaginal atrophy, vaginal stenosis, active vaginitis, hymenal stenosis, depression, use of any medicines, and alcohol and/or any chemical substance addiction^(5,6,7)^.

Along with the changes in human sexual response model from linear to Basson’s circular model^(8)^, the definition of sexual dysfunction was also changed in the diagnostic and statistical manual of mental disorders-fifth edition criteria. The classification was made simpler by reducing categories into three sections. Female hypoactive desire dysfunction, and female arousal dysfunction were merged into a single syndrome called sexual interest/arousal disorder. Similarly, the formerly separate dyspareunia and vaginismus are now called genitopelvic pain/penetration disorder. Female orgasmic disorder remains in place^(9)^.

Human papilloma virus (HPV) is the most prevalent sexually transmitted disease worldwide^(10)^. The transmission of HPV may also occur through intimate contact without intercourse. An HPV screening program has been conducted in Turkey since 2009. Owing to these screening programs, HPV can also be diagnosed in asymptomatic women. Being an HPV carrier and having anogenital condyloma have been associated with anxiety, depression, and sexual dysfunction in previous studies^(11,12)^. Women who tested positive for HPV may experience feelings of guilt, sadness, stigma, and embarrassment, which make them more concerned about sexual contact^(13)^. The aim of this study was to investigate sociodemographic factors affecting female dysfunction along with HPV screening results.

## Materials and Methods

**Figure 1:**
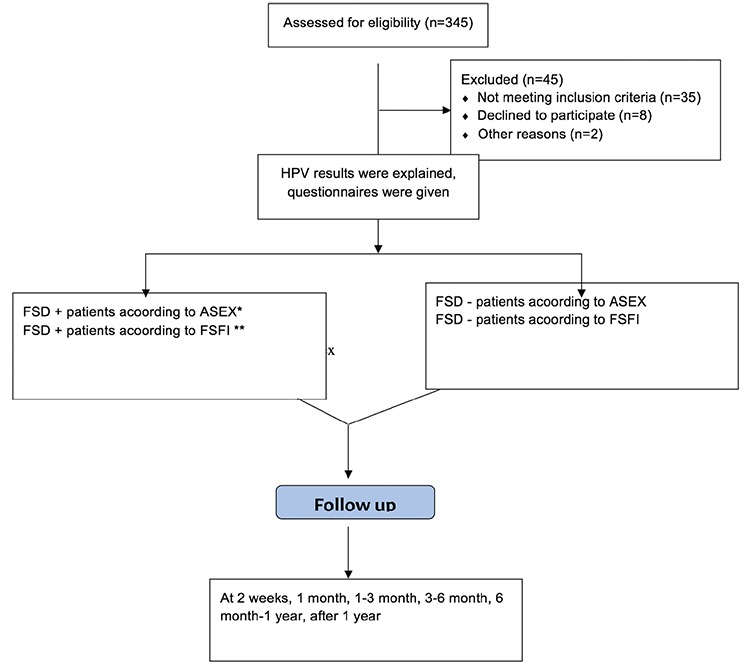
Follow-chart of enrollment FSD: Functional sexual dysfunction, FSFI: Female Sexual Function index, ASEX: Arizona Sexual Experiances

This study was designed as a single-center, prospective, descriptive-cross-sectional study, and was approved by the Dr. Lütfü Kırdar Training and Research Hospital Local Ethics Committee (decision no: 2019/514/148/24). Women who received HPV screening in our hospital between August 1^st^, 2017, and November 1^st^, 2017, were included in the study. The Arizona Sexual Experiences (ASEX) scale and the Female Sexual Function Index (FSFI) were administered to study participants during face-to-face interviews at diagnosis, before 2 weeks, 2 weeks-1 month, 1-3 months, 3-6 months, 6 months-1 year, and after 1 year. Each participant provided written informed consent. A total of 345 women aged 18 to 70 years who were referred for routine gynecologic exams with normal cervical smear results were included in the study. The exclusion criteria were pregnancy; lactation; anogenital lesions; cancer; chronic systemic diseases; infertility; any conditions that might lead to sexual dysfunction including vaginismus, vaginal atrophy, vaginal stenosis, active vaginitis, and hymenal stenosis; depression; use of any medicines; and alcohol and/or any chemical substance addiction. With all these evaluations, 300 patients were included and 45 patients were excluded from the study. The enrollment of the study was showed in Figure 1. The FSFI was developed by Rosen et al.^(14)^ in 2000 to assess female sexual functioning. The validity and reliability of the Turkish version of the questionnaire has been established in a validation study^(15)^. It is a 19-item inventory comprising six domains: desire, arousal, lubrication, orgasm, satisfaction and pain. This questionnaire reflects sexual functioning over the past one month based on subscores from six subscales and the FSFI total score. Subscale scores and the FSFI total score are calculated according to a scoring system developed by the investigators who developed the questionnaire. Domain scores are calculated by summing individual items in a given subscale and multiplying the sum by corresponding domain factor indicated in the relevant table, and the overall FSFI score is calculated by summing all subscale scores. In this study, response options were provided and individual subscale scores were calculated on a Likert-type response scale. A total score less than 26.55 indicates risk for sexual dysfunction^(16)^. All questionnaires were reviewed in order to identify any possible inconsistency.

ASEX is a self-reported questionnaire that was developed by McGahuey et al. ^(17)^ to assess changes in sexual functioning and sexual dysfunction in patients on psychotropic medications. The validity and reliability of the Turkish version of the questionnaire has been established in a study conducted by Soykan. Female and male versions of ASEX are available. It is a five-item scale and items quantify sex drive, psychological arousal, physiological arousal, ability to reach orgasm, and satisfaction from orgasm, respectively. Each item is rated from 1 to 6 with possible total scores ranging from 5 to 30, sexual dysfunction is defined as total scores of 19 or more, or 5 or more on any item, or 4 or more on three items and strongly correlates with clinically defined sexual dysfunction^(18,19)^. Sociodemographic data including age, educational attainment, marital status, household income, and number of children were recorded. Etiologic factors related to FSD were investigated via the ASEX and FSFI questionnaires. Also, the questionnaire subscores of the patients were investigated in terms of HPV screening results. The patients were followed up in order to clarify the effect that could arise afterwards.

### Statistical Analysis

The SPPS 20.0 software (IBM Corp. Released in 2011. IBM SPSS Statistics for Windows, Version 20.0. Armonk, NY: IBM Corp.) was used to analyze the study data. Data were presented as mean ± standard deviation, median (minimum-maximum), percentages, and frequency of variables. Repeated measures of analysis of variance (ANOVA) was analyzed using Mauchy’s Sphericity test and Box’s test of equality of covariance matrices. For comparisons of means, repeated measures ANOVA was used. If parametric tests (factorial design for repeated measures analysis) did not provide the preconditions, Greenhouse-Geisser (1959) correction or Huynh-Feldt (1976) correction or the Friedman test was used for corrections to the degrees of freedom. The corrected Bonferroni test was used for multiple comparisons. Normality and homogeneity of variances were prerequisites to analyze variables using the Shapiro-Wilk and Levene tests. In the analysis of data, the independent Samples t- test (Student’s t-test) was used in comparisons between two independent groups, if prerequisites were met, and the Mann-Whitney U test was used if prerequisites were not met. In comparisons among three or more groups, One-way ANOVA and Tukey’s honestly significant difference multiple comparisons test were used if prerequisites were met, and the Kruskal-Wallis or Bonferroni-Dunn’s multiple comparisons tests were used if prerequisites were not met. Fisher’s exact test and the chi-square test was used to analyze categorical data. When the expected frequencies were less than 20%, the Monte Carlo simulation method was used to include these frequencies in the analysis. The statistical significance level for these tests was set at p values of <0.05 and <0.01.

## Results

A total of 300 women who met the inclusion criteria were included in the study. Cervical cytology samples were obtained. In the follow-up, results were explained to the patients and the ASEX and FSFI scales were administered to participants during this visit and follow-up visits at week 2, week 4, month 3, month 6 and month 12, thereafter. Study participants were stratified based on sociodemographic characteristics and the results of cervical cytology for HPV. The mean age of participants was 42 (range, 22-70) years. Sociodemographic Characteristics scale scores and smear results for HPV are summarized in Table 1.

The assessment of factors affecting total ASEX scale scores of the participants revealed that being aged 45 years and older (p=0.016), marital status (p=0.03), being employed (p=0.01), parity (p=0.011), and low income and high income levels (compared to middle income levels) (p=0.023) significantly increased the prevalence of sexual dysfunction (Table 2). Testing positive for HPV and educational attainment were not found to have statistically significant effects on ASEX scores. The assessment of the effects of sociodemographic characteristics and testing positive for HPV on FSFI revealed that FSFI scores were significantly lower and sexual dysfunction was more common among working women (p=0.006). Age was found to significantly affect total FSFI scores and sexual dysfunction was more common among women aged 20 to 45 years than in women aged over 45 years (p=0.006). In the assessment of participants who tested positive for HPV, total FSFI scores were significantly higher in parous women compared with non-parous women (p=0.027) (Table 2). Income level, educational attainment, and marital status did not significantly affect FSFI scale scores.

The assessments on whether testing positive affected FSFI subscores revealed no differences between women who were HPV-positive and HPV-negative in terms of desire (p=0.670), arousal (p=0.670), lubrication (p=0.490), orgasm (p=0.880), satisfaction (p=0.850), and pain (p=0.380) subscale scores (Table 3).

The comparisons between ASEX scores or FSFI total/subscores at diagnosis, week 2, week 4, month 3, month 6 and month 12 time points revealed that neither ASEX scores nor FSFI total/subscores differed significantly with time from diagnosis of HPV (Table 4).

## Discussion

The HPV carrier incidence was 62.3% in our population, which is much higher than in older studies (17.9%), which might be associated with this study being conducted in a tertiary center. Also, previous studies were conducted in a different region of Turkey and 8 years ago^(20)^. Demir et al.^(21)^ reported an HPV incidence of 31.8% among women aged 25-29 years and a decrease with age. Unlike other studies, this study could not demonstrate any potential effects of a diagnosis of HPV on sexual functioning. Furthermore, time from diagnosis had no effects on sexual functioning. Unlike our study, Ferenidou et al.^(22)^ concluded that women who were diagnosed as having HPV experienced negative feelings and a reduction in sexual desire. According to the results of a study conducted by McCaffery et al.^(23)^, testing positive for HPV might lead to anxiety, and necessary education should be provided to HPV-positive women after sharing test results. The results in our studies may be explained by the exclusion of women with anogenital lesions and abnormal Pap-smear results at screening. In addition, this study was the first and single study investigating the effects of a diagnosis of HPV and time from diagnosis on sexual functioning, and the differences in the results compared with studies conducted abroad might be explained by cultural differences. Psychosocial effects of HPV-linked diseases and abnormal cytology results have been demonstrated in previous studies^(24)^. Previous studies showed that even positive screen results for HPV might have psychosocial consequences. These effects could cause anxiety, stress or reluctance to engage in sexual activity. Independently from cervical cytology results, it has been demonstrated that a positive HPV test alone might make women feel bad about sexual relationships^(25)^. In our study group, sexual dysfunction scores at the time of detection of HPV positivity were examined and these scores were compared. However, it would be more meaningful to compare the scores of the same patient before the HPV test and the scores after receiving the HPV test results. This is the most important limitation of our research.

Female sexuality has been increasingly investigated over recent years. New questionnaires and algorithms have been developed to ensure the objectivity of assessments^(26,27)^. The FSFI is widely used to investigate female sexual functioning and received a wide acceptance; the validity of the Turkish version has been established^(28)^. In this study we also used the ASEX questionnaire in order to have more accurate results.

Previous studies investigated the associations between advanced age and sexual functioning and revealed similar rates of sexual problems between older women and younger women who were referred for routine gynecologic exams^(29)^. It has been reported that menopausal status has no negative impact on sexual functioning; however, stress, previous sexual experiences, and general health status are more important determinants of sexual health^(30)^. In this study, we also observed that sexual functioning was better in older women compared with younger women. Reduced responsibilities and mother and child dependency could explain this observation. The increased prevalence of sexual dysfunction among women with children compared with woman without children also provides further support to this assumption. In the literature, several studies reported that the first delivery and breastfeeding might cause sexual dysfunction^(31)^. A study assessing postpartum sexual functioning was conducted in the Netherlands and reported that older age at delivery was related to better sexual functioning^(32)^.

In the literature, no association between educational attainment and sexual functioning has been clearly established^(33)^. In our study, educational attainment had no significant effects on ASEX total scores and FSFI total scores. In a study conducted by Laumann et al.^(34)^, the prevalence of sexual dysfunction and possible predictors of sexual dysfunction were investigated and a negative correlation was found between higher educational attainment levels (college graduates) and the prevalence of sexual dysfunction. In our study, no significant association was found. These results may be explained by the fact that sex education is not included in the school curriculum in our country and any information is hearsay or obtained by individuals on their own.

A literature search on the effects of income levels on sexual functioning revealed that several publications reported an association between lower income levels and a higher prevalence of sexual dysfunction, whereas others reported the opposite^(35)^. In this study, sexual dysfunction occurred less in the middle income group than in the other income groups. The assessment of income levels in combination with other variables indicated that sexual dysfunction was more likely among those with pre-existing depression and/or lower educational attainment, in addition to having a low income level. In previous studies, sexual dysfunction has been reported in more than one-third of women with more than 11 years of schooling^(36)^. However, there are also contradictory studies in the literature^(37)^. It is possible that we could not clearly demonstrate such differences because our population was not homogenous in terms of income levels. These differences might be clearly demonstrated in studies with larger and homogenous study samples. The effects of employment status on sexual dysfunction remained unclear in a recent study conducted in 2018, whereas partner’s unemployment was suggested as a risk factor^(38)^. Sexual dysfunction was more prevalent in working women in our study. However, the nature of work and working hours were not investigated in our study, and potential effects of employment status on sexual functioning may be more clearly defined by taking these factors into consideration.

This study is important because it is the first to investigate the effects of testing positive for HPV on sexual functioning in Turkish woman over an extended follow-up period. Furthermore, bias was avoided by using two different questionnaires.

## Conclusions

Educational attainment, socioeconomic status, age, employment status, and marital status were found to have statistically significant effects on sexual functioning. Sexual functioning was affected by neither HPV test results (positive/negative) nor time from diagnosis.

## Figures and Tables

**Table 1:**
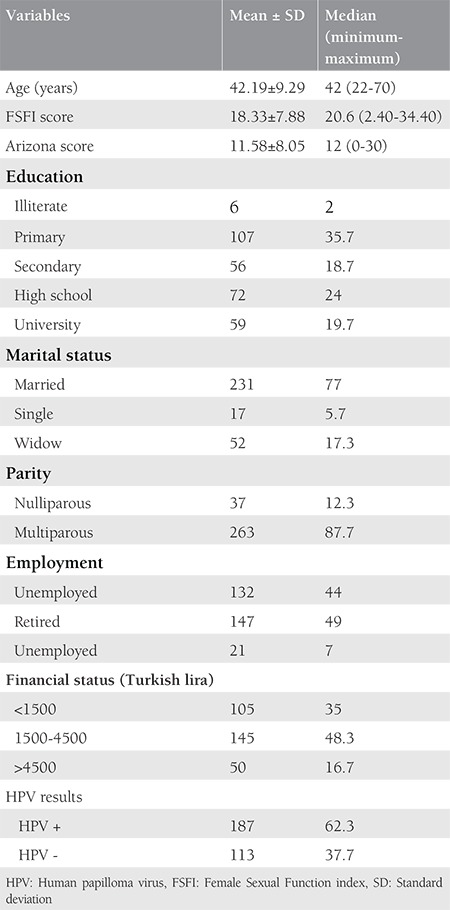
Socio-demographic and maternal characteristics of participants

**Table 2:**
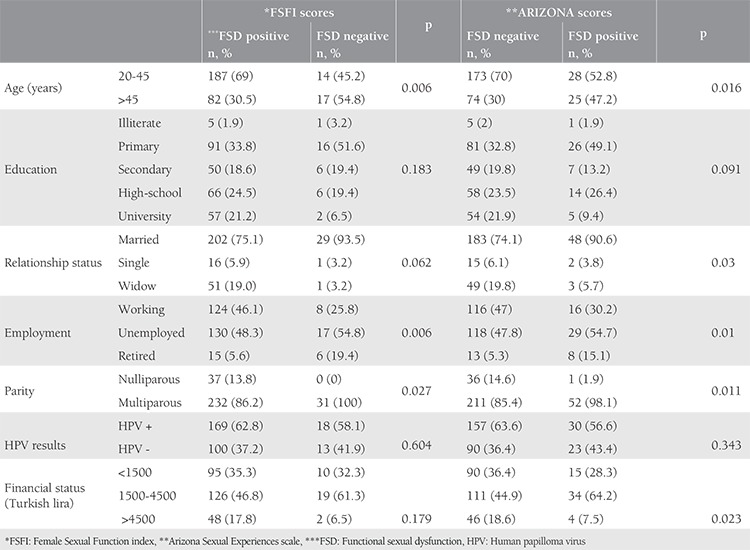
Relationship between sociodemographic factors and human papilloma virus results of the patients and functional sexual dysfunction according to Female Sexual Function index and ARIZONA scores

**Table 3:**
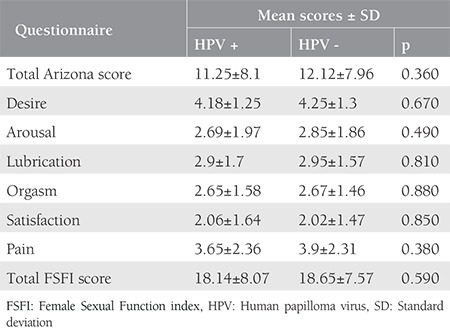
Female Sexual Function index and Arizona comparative scores among women with and without human papilloma virus

**Table 4:**
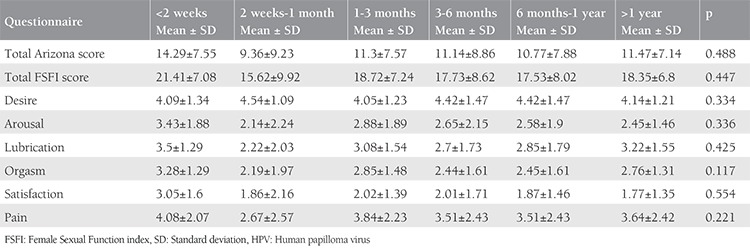
Comparison of Female Sexual Function index and Arizona scores in time after testing positive for human papilloma v
